# Hydrometeorology and geography affect hospitalizations for waterborne infectious diseases in the United States: A retrospective analysis

**DOI:** 10.1371/journal.pwat.0000206

**Published:** 2024-08-14

**Authors:** Victoria D. Lynch, Jeffrey Shaman

**Affiliations:** 1 Department of Environmental Health Sciences, Columbia Mailman School of Public Health, Columbia University, New York, New York, United States of America,; 2 Columbia Climate School, Columbia University, New York, New York, United States of America

## Abstract

Meteorology, hydroclimatology, and drinking water infrastructure influence the transmission of waterborne infectious diseases in the United States, but their roles are not well-understood and may vary by pathogen type or geographic region. Waterborne pathogens can cause severe intestinal, respiratory, or systemic infections in vulnerable people. Identifying the mechanisms that underlie contamination events and disease transmission is particularly important given that climate change may lead to more extreme floods, droughts, and seasonal precipitation. The association of meteorological variables, drinking water source, geographic region, and location (rural/urban) with hospitalizations for 12 waterborne bacterial, parasitic, and viral infections was examined using 12 years of hospitalization data from 516 hospitals in 25 states. A multimodel inference approach was used to identify the most highly-weighted explanatory variables and these were included in a generalized linear mixed model (GLMM) framework. There was a 16% (95% CI: 8%−24%) decrease in hospitalizations for the bacterial pathogen group in urban compared to rural areas; for *Campylobacter*, specifically, there was a 31% (95% CI: 9%−53%) decrease in urban areas, a 27% (95% CI: 6%−48%) decrease associated with drinking water from surface water sources, and an 11% (95% CI: 4%−17%) increase with a 1-standard deviation (SD) increase in runoff. Parasitic hospitalizations increased 9% (95% CI: 4%−15%) with a 1-SD increase in precipitation, predominantly driven by *Cryptosporidium* hospitalizations. Legionnaires’ disease increased 124% (95% CI: 90%−157%) with a 1-SD increase in soil moisture. Associations between hospitalization rates and meteorological conditions, location, and drinking water source varied among the specific pathogens; the pathogen-group level analyses masked several of these findings and were largely uninformative. Precipitation, runoff, and rural locations were positively associated with hospitalizations for some enteric bacterial and parasitic infections; conversely, hospitalizations for biofilm-forming bacterial infections were associated with soil moisture and hospitalization rates were higher in urban areas.

## Introduction

Waterborne infectious disease is a persistent problem in the United States, where an estimated 7,150,000 cases occur annually despite drinking and recreational water regulations, and sanitation infrastructure [1]. Waterborne pathogens transmitted via contaminated environmental or drinking water can cause severe respiratory or gastrointestinal infections, particularly among vulnerable groups [2, 3]. Drinking water and wastewater treatment substantially reduces the burden of disease but these systems are still vulnerable to contamination, a problem that will likely intensify in conjunction with aging infrastructure [4].

The effect of meteorological conditions on waterborne diseases may differ among the causative pathogens, which include biofilm-forming bacteria, enteric bacteria, protozoa, and viruses. Important characteristics related to transmission, including their reservoirs and ability to persist in the environment, vary among these groups [5–10]. The proportion of infections attributable to waterborne transmission, rather than foodborne or person-to-person, also substantially varies among them [11]. The biofilm-forming bacteria (e.g. Nontuberculous mycobacteria, *Pseudomonas*, *Legionella*) are natural inhabitants of water and transmission is almost entirely waterborne [12]. They are abundant in environmental water and thrive in biofilm communities that colonize water distribution systems and water storage facilities [13–15]; *Legionella*, in particular, is associated with outbreaks linked to plumbing systems [16]. When inhaled, the biofilm-forming bacteria can cause opportunistic infections among immunocompromised, elderly, or hospitalized people [13, 17, 18]. As these pathogens are ubiquitous in drinking water [19, 20] and household plumbing [12], and may also cause a notable proportion of community-acquired pneumonia [21].

Pathogens that cause gastrointestinal illness are often introduced into the environment through human or animal waste and, while not natural inhabitants of water like the biofilm-forming bacteria, can persist in soil and waterbodies for months [8, 22–24]. These bacteria and protozoa share many of the same animal reservoirs but differ in their primary transmission routes; protozoa are predominantly waterborne, though foodborne is also common, whereas a small fraction of enteric bacteria is thought to be waterborne [11]. The origin of many foodborne cases is unknown, however, and water may play a critical and under-recognized role in foodborne transmission [25]. Agricultural runoff and irrigation water are often pathogenic and can contaminate crops leading to foodborne outbreaks that are at least partially, or indirectly, driven by surface water [26, 27]. The seasonality of both enteric bacterial and protozoal infections suggests that meteorological factors influence the contamination events necessary for transmission [28, 29].

Intense precipitation, flooding, and drought affect the concentration and dispersal of waterborne pathogens. Floods mobilize pathogens in sediment, soil, and water and overwhelm sanitation infrastructure so that sewage circumvents treatment [30]. Flooding after prolonged dry periods is of particular concern. Low-flow conditions during droughts can increase pathogen concentration in water distribution systems; this pathogenic water is then flushed out with rapid flood-driven inflow [31, 32]. Most previous research has focused on non-specific gastrointestinal infectious disease and has found positive associations with flooding [33], precipitation [34], dry periods [32, 35], and temperature [36, 37], which affects the survival of some pathogens in the environment. Some pathogen- and location-specific studies have found more inconsistent associations, however, which indicate that the effect of meteorological variability is not uniform across regions or pathogens [38, 39].

The contamination events necessary for transmission are governed by dynamic interactions among hydroclimatology, land use, and water infrastructure. Meteorological conditions that lead to contamination in one setting may have no effect in regions with different hydroclimatology [40–42]; for example, Cryptosporidiosis has been found to increase with precipitation and temperature in tropical and temperate climates [43], but exhibits no seasonality or association with meteorological variables in arid regions [38]. Environment-disease dynamics can vary even within a small geographic area; precipitation has been positively associated with *Campylobacter* and *Salmonella* bacterial infections in low-lying coastal areas, but not inland regions, within a single state in the US [44–46].

Drinking water sources from both groundwater [47, 48] and surface water [49] are susceptible to contamination but meteorological drivers, exposure routes, and pathogens may vary by location [2, 47, 50], particularly between urban and rural areas [51]. In places with extensive impermeable surfaces, namely cities or regions experiencing drought, precipitation can lead to flash floods that cause sewage by-passes or combined sewer overflows (CSOs) [52]; this wastewater is highly pathogenic [53, 54] and can contaminate surface water sources used for drinking water [55, 56]. In agricultural regions with large drainage basins, however, exposure is often driven by snowmelt [53], which generates standing water in fields and runoff polluted with animal waste [48]. Floodwater carrying pathogens from soil, including biofilm-forming bacteria, and from livestock fecal matter can contaminate drinking water from groundwater sources through direct contact or infiltration [48, 57, 58].

In this study, we examined the effect of meteorological variables on hospitalizations for waterborne infectious diseases and whether these associations were influenced by drinking water source, location (rural/urban), and region. To account for the potential role of floods in driving transmission, we included multiple flood-indicator variables, such as soil moisture and surface runoff, to capture the various flood types (e.g. river floods, flash floods) that occur in the US. We assessed these associations for bacterial, protozoal, viral, and biofilm-forming pathogen groups, and for each pathogen independently, to determine whether environment-disease dynamics were consistent among pathogens with similar biology. Previous research has examined the effect of precipitation or temperature on cases, but these studies have focused on nonspecific diarrheal illness, narrow geographic regions, or on outbreaks. Waterborne infectious diseases will become a more pressing public health challenge as climate change leads to more severe floods and droughts [59]. A thorough understanding of contamination mechanisms is necessary to identify communities at risk for waterborne illness and to develop effective interventions.

## Methods

### Data

#### Hospitalization data.

The Centers for Disease Control and Prevention (CDC) has identified 17 waterborne diseases or syndromes that are endemic to the US and can cause severe illness [1]. In this analysis, we used the National Inpatient Sample (NIS) from the Healthcare Cost and Utilization Project (HCUP) to identify hospitalizations for 12 enteric or respiratory diseases that caused by waterborne bacterial, protozoal, and viral pathogens. We did not include Otitis externa (ear infections) or *Vibrio* infections, which are often driven by foodborne exposure to contaminated seafood or wound exposure to seawater [60]. We also included hospitalizations for unspecified intestinal amoebic and protozoal infections which, while not included in CDC surveillance data, are waterborne pathogens can cause severe disease [61–63]. We identified infections by ICD-9 code and found the monthly hospitalization count for each of the 12 pathogens at every hospital between 2000 and 2011. We restricted our analysis to hospitals that contributed at least 4 years of data to the NIS dataset, provided monthly counts of hospitalizations, and reported their exact geographic location. We excluded one hospital in Denver, Colorado, that specialized in respiratory infections, including those caused by the biofilm-forming pathogens included in this analysis, and treated patients from across the US. Since the NIS data are de-identified and in a publicly available dataset, Columbia University’s Human Research Protection Office does not consider this to be research with human subjects and thus does not require an IRB review.

In the primary analysis, we also restricted hospitals to those that had at least 10 hospitalizations for waterborne infections during the study period. Hospitals with low case counts may be in areas where contamination is rare, and the few treated cases are travel-related, or may indicate that the hospitals do not test for specific waterborne pathogens. As a sensitivity analysis for this exclusion criteria, we repeated the analysis using several case count thresholds. We created subsets of our hospitalization data containing hospitals with at least 1, 5, 15, and 20 cases of waterborne disease during the study period; all of the analyses were repeated with these case threshold datasets.

Hospitals were categorized by location (rural/urban) and size (number of hospital beds) according to the definitions used by HCUP. Prior to 2004, urban hospitals were those within Metropolitan Statistical Areas (MSAs), as defined by the US Census Bureau based on 1990 Census data, and rural hospitals were those outside MSAs. From 2004 to 2011, Core Based Statistical Areas (CBSAs) defined by the US Census Bureau were used to determine location; hospitals within ‘Metropolitan’ or ‘Division’ CBSAs were considered urban while those in ‘Micropolitan’ or ‘Rural’ CBSAs were rural. Hospital size was determined by the number of hospital beds given the hospital’s geographic region, location, and teaching status (teaching hospital or non-teaching hospital).

#### Meteorological data.

Precipitation, soil moisture, surface runoff, and temperature data were obtained from the NASA/ NOAA North American Land Data Assimilation System 2 (NLDAS-2) forcing dataset and were aggregated from hourly temporal resolution to mean monthly values for each hospital location. Correlation among the meteorological variables was assessed; if multiple variables were highly correlated (r > 0.8), a single variable was selected for further analysis.

#### Drinking water data.

Drinking water data were extracted from the Safe Drinking Water Information System (SDWIS) for the community water system (CWS) that served each hospital. The coordinates of the center of each CWS were matched to the closest hospital by latitude and longitude. SDWIS includes a binary variable to indicate if the CWS is considered to have groundwater or surface water in addition to other water system variables. Primary water source, ownership of water system, and implementation status of source water protection measures data were also extracted from SDWIS for each CWS; correlation among water system variables was assessed as described for the meteorological variables.

### Qualitative seasonality assessment and trend analysis

We categorized the hospitalizations for waterborne illnesses into “bacterial”, “parasitic”, “biofilm-forming”, and “viral” pathogen groups. The biofilm-forming pathogens are bacterial, but distinct from the other bacterial pathogens in that they are natural inhabitants of environmental water; this group includes hospitalizations for Legionnaires’ disease, respiratory and intestinal *Pseudomonas* infections, and Nontuberculous mycobacteria infections. The bacterial pathogen group includes *Salmonella*, *Campylobacter*, *Shigella*, and *Escherichia coli* infections. The parasitic pathogens are *Cryptosporidium*, *Giardia*, and multiple species of amoeba and protozoa (not specified in the NIS); finally, the only viral pathogen is Norovirus.

We assigned the hospitals to geographic regions according to United States Geological Survey (USGS) categories, with slight modifications to prevent single states from being the only representative in a region. The pathogen group and geographic region variables were used to assess how seasonality and trends in hospitalizations varied throughout the US during the study period. For each hospital, we calculated monthly hospitalization rates per 10,000 hospitalizations for each waterborne pathogen. We averaged monthly hospitalization rates by pathogen group, geographic region, and month to qualitatively assess if there was a distinct seasonality.

We also averaged monthly hospitalization rates by only geographic region and used the Mann-Kendall test to examine the differences in interannual trends between 2000 and 2011 across the US. We repeated the qualitative seasonality and trend analyses with the pathogen-specific hospitalizations to evaluate the consistency within the pathogen groups.

### Statistical analysis

We modeled the association between waterborne disease hospitalization rates and meteorological, drinking water source, and location variables using a negative binomial generalized linear mixed model (GLMM) framework to account for overdispersion in the hospitalization data. Drinking water source was included as a binary variable (groundwater or surface water), and location variables included terms for geographic region (New England, Mid-Atlantic, Central Midwest, North-Central Midwest, Mountain, and Pacific) and hospital location (rural or urban). The model included a term for year and annual sine and cosine terms to adjust for secular and seasonal trends, respectively. We also included a random intercept for each hospital to account for hospital-specific differences in admission and testing policies. Hospital- and month-specific discharges were used as an offset to obtain the rate of hospitalizations; we present all results from the statistical analysis as percent changes in hospitalization rates. We modeled hospitalization rates for each pathogen separately and as pathogen-type groups. The GLMM model structure, including a description of each term in the equation, and its assumptions are described in the supplementary material (S1 Text).

Multimodel inference was used to compare models with all combinations of the standardized meteorological variables, drinking water source, geographic region, and hospital location and to determine the importance weight of each explanatory variable. The candidate models varied only in these variables, but otherwise had the same structure. We used log likelihood and the number of parameters to calculate the Akaike weight for each model and ranked them by weight [64]. The top models were the smallest number of models whose weights summed to 0.90 or greater.

Next, the top models were used to calculate the relative importance weight for the meteorological, drinking water, and location variables. For each of these variables, the relative importance was determined by summing the Akaike weights for all of the top models that included the given variable. The most important predictor variable was estimated to be the variable with the largest relative importance weight [65]. Finally, the top model was identified as the candidate model with the most highly weighted variables (those with relative importance weights ≥ 0.5); this process was repeated for each pathogen group and specific pathogen. Collinearity was assessed by variance inflation factor (VIF); models with a VIF less than 5 were considered unaffected by collinearity [66]. Cross-validation was performed by removing 20% of the data and conducting multimodel inference on the remainder; this process was iterated 1,000 times to evaluate the consistency of the weights and effect estimates, and to compare these results to the top full models. These analyses were repeated for each case-count threshold.

### Sensitivity analyses

The NIS includes the location of the reporting hospital, but not case residential locations. To address the possibility of misclassification bias, given that flood data are associated with the location of a hospital, we matched the hospitals to Hospital Service Areas (HSA) provided by the Dartmouth Atlas of Healthcare [67]. The HSA is the catchment area for each hospital and includes the zip codes where most Medicare patients receive care from a given hospital. We repeated the analyses using flood data associated with the HSA catchment area, instead of the hospital location, as a sensitivity analysis to assess the consistency of our findings.

## Results

### Waterborne disease hospitalizations

**Fig 1.**
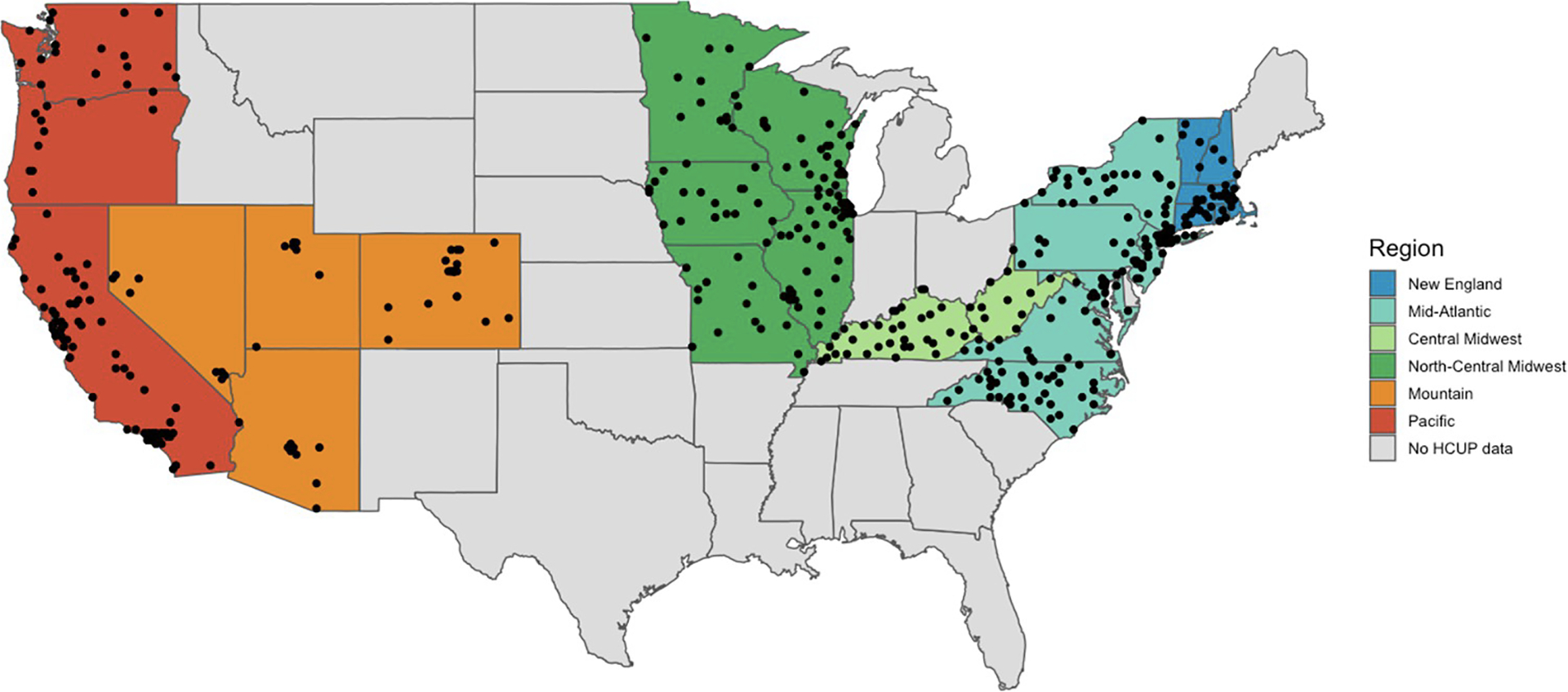
Hospitals included in the analysis. 516 hospitals (black circles) in the HCUP dataset met the inclusion criteria and had a minimum of 10 total hospitalizations for at least one of the waterborne infectious diseases. The dark gray states are those that did not provide monthly geolocated data or did not report to HCUP. Map was created in R with the rnaturalearth package, which uses an base maps from Natural Earth that are in the public domain [68].

There were 57,335 hospitalizations for waterborne disease between 2000 and 2011 from 516 hospitals in the United States (Fig 1). The biofilm-forming pathogens comprised nearly 81% of all hospitalizations for waterborne illnesses, with 66% of hospitalizations due to respiratory *Pseudomonas* infections alone (Table 1). Apart from the *Pseudomonas*-related hospitalizations, the most common causes for hospitalization were Nontuberculous mycobacteria (NTM) infection (9.6%), *Salmonella* infections (8.0%), and Legionnaires’ disease (4.1%) (Table 1).

Hospitalization rates for enteric and biofilm-forming bacterial pathogens were significantly higher in areas that used groundwater as a drinking water source instead of surface water (Table 2); parasitic hospitalization rates were slightly elevated as well, but the difference was insignificant (p = 0.97). However, the pathogen groups did not accurately reflect the pathogen-specific differences in hospitalizations by drinking water source (S1 Table). Cryptosporidiosis hospitalization rates were almost three times greater in groundwater areas compared to surface water while Giardiasis rates were slightly higher in the latter (S1 Table). Among the enteric bacteria, Campylobacteriosis and *E*. *coli* hospitalization rates were much higher in groundwater while Salmonellosis and Shigellosis were evenly split between drinking water categories (S1 Table).

Hospitalization rates for enteric and biofilm-forming bacteria were also significantly higher in areas with privately owned CWSs, and in state-owned systems just for biofilm hospitalizations (S2 Table). Bacterial and parasitic rates did not substantially vary by primary water source (i.e. purchased groundwater, groundwater under influence of surface water, etc.) or by whether source water protection had been implemented (S2 Table). Among biofilm-forming pathogens, however, hospitalization rates were much higher in areas that relied on purchased groundwater and that had not implemented source water protection measures (S2 Table).

Hospitalization rates for all of the pathogen groups were greater in small and rural hospitals, especially for the parasitic infections (Table 2). The pathogen-specific analysis demonstrated, however, that Legionnaires’ disease hospitalization rates were higher in urban areas unlike the other pathogens in its group (S1 Table). There were starker differences among group hospitalizations by geographic region; they were highest in the North-Central Midwest and Central Midwest regions for parasitic infections and in the Mountain and Pacific regions for biofilmrelated infections. Hospitalization rates for enteric bacterial infections were relatively consistent across the geographic regions, though slightly higher in the North-Central Midwest (Table 2). An estimated 0.4% of all Norovirus cases lead to hospitalizations [1] and there were few in the dataset. Among the hospitals that reported cases, Norovirus hospitalization rates were greater in medium-sized, rural hospitals and in the Pacific states (Table 2). These findings were not skewed by the specific HCUP hospitals included in the analysis; the number, size, and geographic breakdown of the hospitals was relatively consistent across pathogen group, though hospitals contributing to the parasitic pathogen group were disproportionately located in the North-Central Midwest and less likely to be located in the Pacific compared to the other geographic regions (S3 Table). Most of the hospitals in the analysis were large facilities and located in urban areas with the exception of the hospitals restricted by *Pseudomonas* case thresholds; among these hospitals, which had at least 10 *Pseudomonas* infections, 30.4% were in rural areas and 56% were small- or medium-sized (S4 Table).

### Qualitative seasonality assessment and time series trends in hospitalization rates

**Fig 2.**
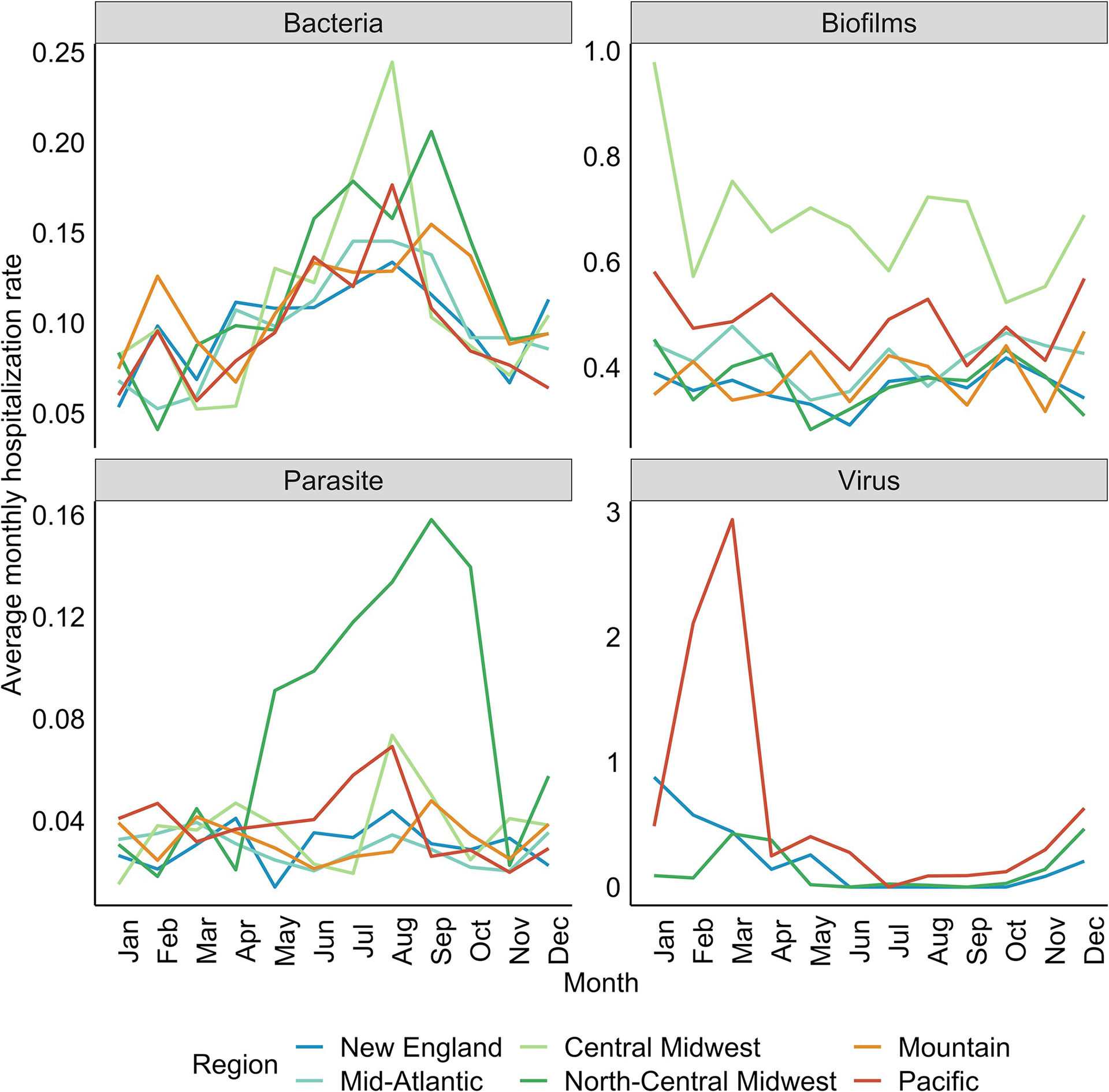
Seasonality of hospitalization rates by pathogen group and geographic region. Average monthly hospitalization rates per 10,000 hospitalizations for bacterial infections peaked between July and September for all regions; parasitic hospitalization rates exhibited a seasonality similar to bacterial infections but only in the Midwest and Pacific regions. There was no clear seasonality to hospitalizations for biofilm-related infections and Norovirus hospitalizations peaked during winter months, though data were limited to only 13 hospitals in New England, North-Central Midwest, and Pacific states.

The seasonality of waterborne disease hospitalization rates varied by pathogen group and region in the United States (Fig 2). The bacterial pathogens exhibited the most consistent seasonality with hospitalizations peaking between July and September in all geographic regions (Fig 2A). During peak months, the average hospitalization rate for bacterial infections was greatest in the Central and North-Central Midwest compared to the other regions; this difference was not evident throughout the rest of the year, when hospitalizations for bacterial infections were comparable among the geographic regions. These findings were consistent across the specific bacterial pathogens, though *Campylobacter* hospitalizations peaked earlier in the year in all geographic regions (S1 Fig).

Seasonality for the parasitic pathogen-group was driven by hospitalizations for *Cryptosporidium* infections in the North-Central Midwest, which exhibited a sharp increase in May and then consistently increased until September (S2 Fig); in the other geographic regions, peaks occurred throughout the year. There was no seasonality to hospitalizations for any of the other parasitic pathogens (S2 Fig). Amoebic and protozoal hospitalizations were higher in the Mountain and Pacific regions throughout the year, but there were too few cases to assess seasonality.

There was no clear seasonality to hospitalizations for infections caused by biofilm-forming pathogens (Fig 2C), though this group-level analysis obscured the seasonality of specific pathogens. Hospitalizations for Legionnaires’ disease peaked between August and October in all regions except the Pacific states, and for intestinal *Pseudomonas* infections in the late fall and winter (S3 Fig). Finally, the only hospitals that met the 10-case threshold for Norovirus were in New England, the North-Central Midwest, and the Pacific, and hospitalizations peaked between January and March in all of those regions (Fig 2D).

The Mann-Kendall analysis found that between 2000 and 2011 there was no significant interannual change in monthly hospitalization rates for any of the waterborne pathogen groups (S5 Table). This was consistent across the specific pathogens and diseases except for Legionnaires’ disease and Nontuberculous mycobacteria, which increased in New England, Mid-Atlantic, and the North-Central Midwest (S4 Fig). Between 2008 and 2011, biofilm-related hospitalization rates increased in areas served by surface water and slightly decreased in areas that used groundwater for drinking water (S5 Fig); this trend was not evident among the bacterial or parasitic hospitalization rates.

### Associations between meteorological variables and hospitalization rates

**Fig 3.**
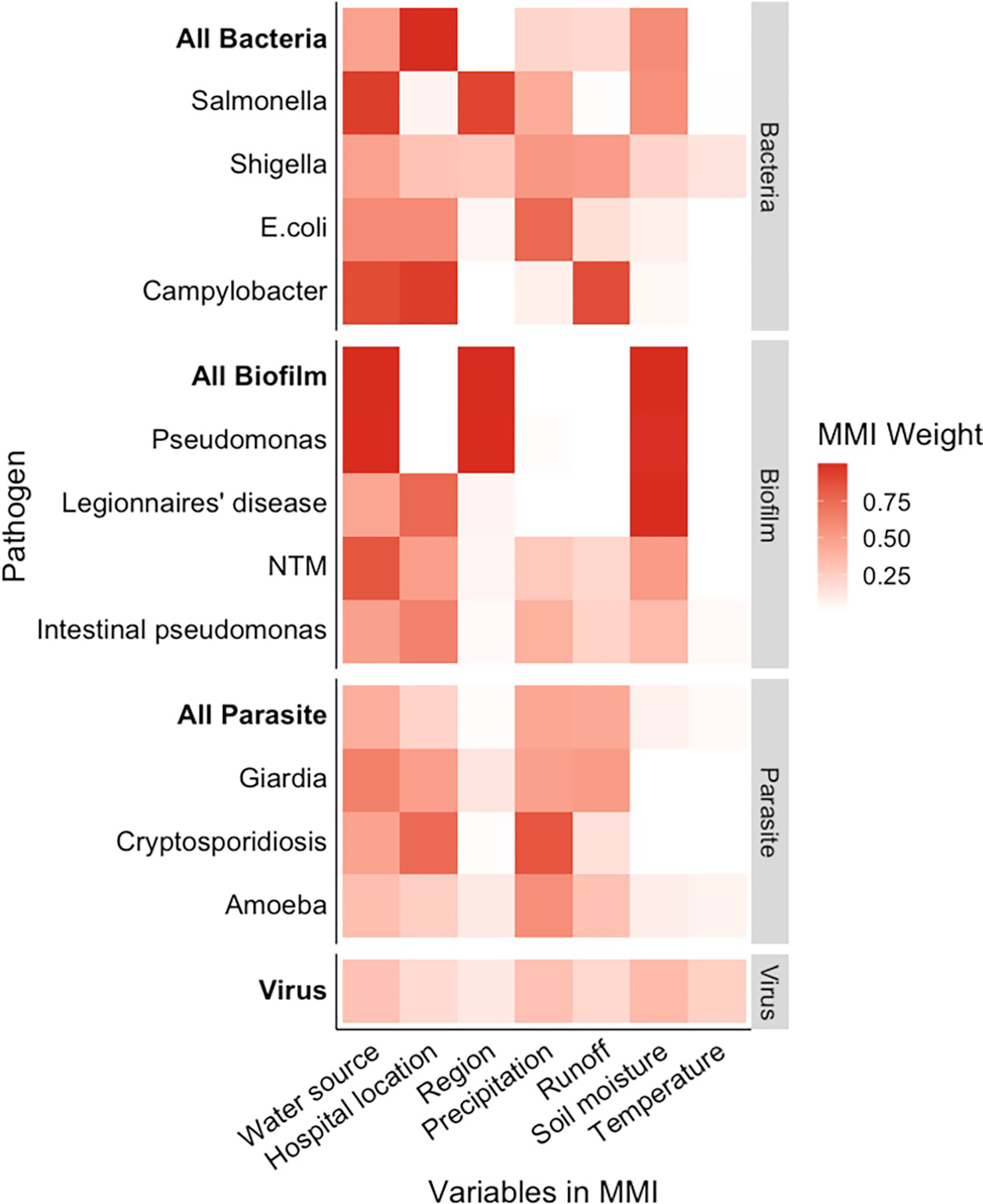
Importance weights identified by multimodel inference. Consistency between the group-level and pathogen-specific weights varied. Drinking water source and hospital location (rural/urban) were highly or moderately weighted in most of the models, but the importance weights for the meteorological variables were inconsistent between the group-level and pathogen-specific models. Runoff was highly weighted for *Campylobacter* while precipitation was moderately weighted for the other enteric bacteria. Soil moisture was highly weighted for most of the biofilm-forming pathogens. Among the parasitic pathogens, only precipitation in the *Cryptosporidium* model was highly weighted.

The most highly weighted meteorological variables identified by multimodel inference varied both among and within the pathogen groups, though drinking water source and hospital location were at least moderately weighted for most of the pathogens (Fig 3). The biofilm-forming group was the most consistent, with soil moisture and drinking water source highly weighted for the overall group and for all of the specific pathogens, other than intestinal *Pseudomonas* hospitalizations. Region was highly weighted only for respiratory *Pseudomonas* while hospital location (rural/urban) was moderately weighted for all of the other biofilm-forming pathogens. Multimodel inference for the bacterial pathogen group also moderately weighted drinking water source and soil moisture, though the latter was due to *Salmonella* hospitalizations (Fig 3). The pathogen-specific models were not well-aligned with the overall model; region was highly weighted only for *Salmonella*, while hospital location was highly or moderately weighted for *Campylobacter*, *E*. *coli*, and *Shigella*. Runoff was highly weighted in the *Campylobacter* model and precipitation was moderately weighted for *E*. *coli* and *Shigella* (Fig 3). Water source, hospital location, precipitation, and runoff were moderately weighted in the parasitic pathogen groups and more highly weighted for *Cryptosporidium* on its own (Fig 3). There were not enough amoeba and protozoal cases to assess the effects of region, hospital location, or drinking water source. Finally, in the Norovirus model none of the explanatory variables had high importance weight.

**Fig 4.**
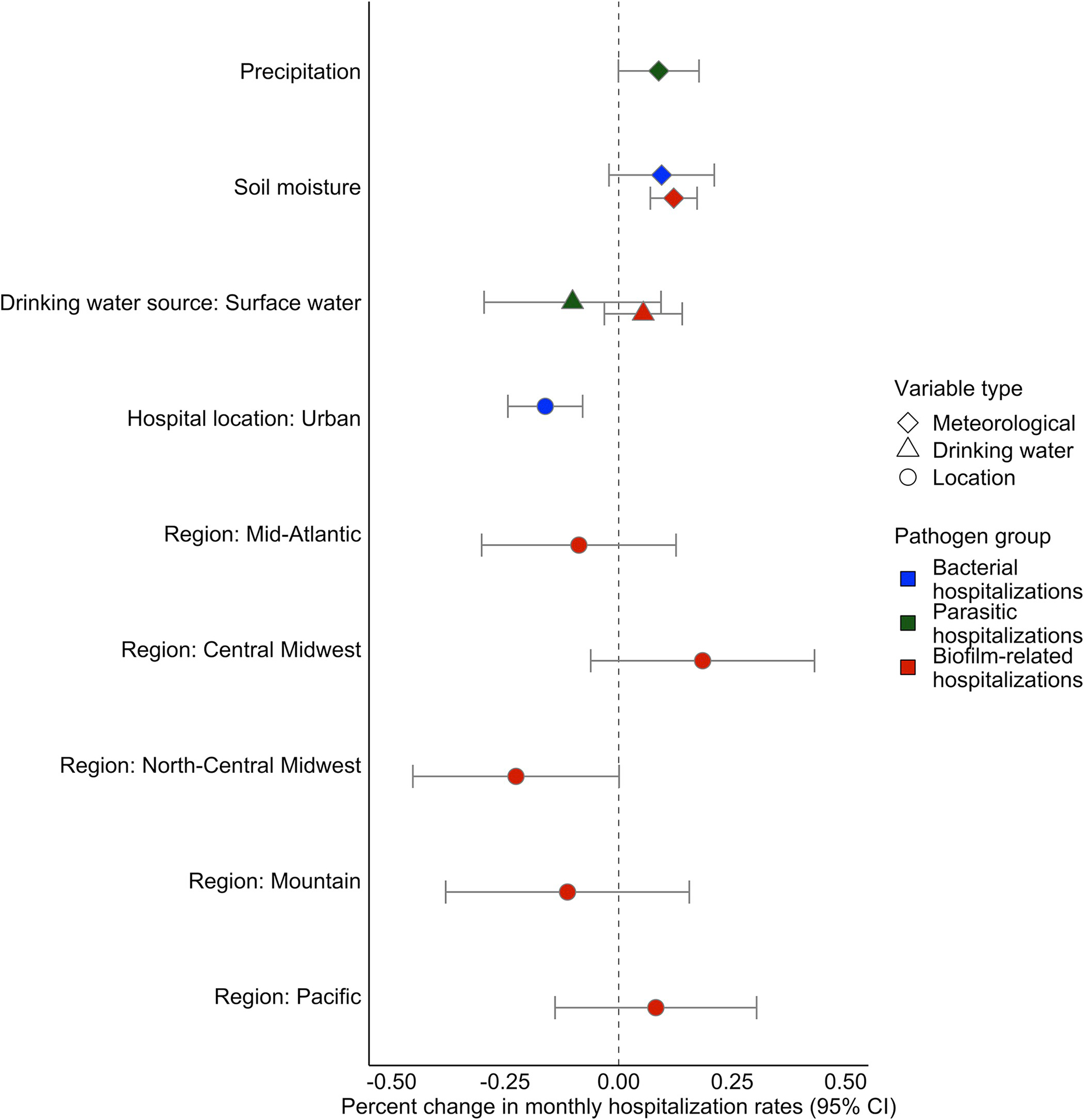
Effect estimates from top model for each pathogen group. There was a 16% decrease in the hospitalization rate for bacterial infections (blue) in urban compared to rural areas and soil moisture was included in the top model, but the positive association was marginally insignificant. Biofilm-related hospitalization rates (red) increased 12% with a 1-standard deviation increase in soil moisture and were greater in areas that used drinking water from surface water sources (Table 2), though this association was marginally insignificant in the model. A 1-SD increase in precipitation was associated with a 9% increase in hospitalization rates for parasitic infections (green).

In the top model identified by multimodel inference, there was a 16% (95% CI: 8% – 25%) decrease in hospitalization rates for the bacterial pathogen group in urban compared to rural locations (Fig 4), which was largely driven by a 31% (95% CI: 9% – 53%) decrease in *Campylobacter* hospitalizations in urban areas (Table 3). *Campylobacter* hospitalization rates also increased 11% (95% CI: 4% – 17%) in association with a 1-standard deviation (SD) increase in runoff and decreased 27% (95% CI: 6% – 48%) in areas that used drinking water from surface water instead of groundwater sources (Table 3). *E*. *coli* hospitalization rates increased in rural areas but decreased 14% (95% CI: −29% – 1%) with a 1-SD increase in precipitation, though these effects were marginally significant.

Hospitalization rates for biofilm-related infections increased 12% (95% CI: 7% – 17%) with a 1-SD increase in soil moisture (Fig 4), but the group-level findings obscured pathogen-specific associations. A 1-SD increase in soil moisture was associated with a 124% (95% CI: 90% – 157%) increase in Legionnaires’ disease and a 9% (95% CI: 4% – 15%) increase in respiratory *Pseudomonas* hospitalizations (Table 3). Drinking water from surface water sources was also associated with an 8% (95% CI: −1% – 17%) increase in respiratory *Pseudomonas*, though the effect was marginally significant. Intestinal *Pseudomonas* hospitalization rates, meanwhile, decreased 62% (95% CI: 2% – 121%) in urban areas, unlike the other biofilm-forming pathogens that were higher in urban locations, and were positively, though not significantly, associated with precipitation.

*Cryptosporidium* and amoebic hospitalization rates exhibited a similar relationship with precipitation, though location and drinking water variables were not included when modeling amoebas because data were too sparse. A 1-SD increase in precipitation was associated with a 22% (95% CI: 1% – 44%) increase in Cryptosporidium and a 24% (95% CI: −2% – 51%) increase in amoebic infections, though these effects was marginally insignificant (Table 3). Norovirus did not have a significant relationship with any of the meteorological variables in either the best model or the average of the top models. The importance weights, best model, and effect estimates were consistent across the hospitalization thresholds (S6 Fig) and hospital service areas.

## Discussion

Hospitalization rates for waterborne infectious diseases were associated with meteorological conditions, location, and drinking water source throughout the United States; however, the strength and direction of the relationships varied among pathogens. Rurality, runoff, and precipitation were associated with some bacterial and parasitic infections that are also common among livestock; conversely, soil moisture had an effect on hospitalization rates for biofilm-related infections and for Legionnaires’ disease, hospitalizations were higher in urban areas. In general, the pathogen groups obfuscated important pathogen-specific associations and were ineffective at identifying trends.

Pathogen-specific water quality monitoring is onerous and expensive [69], and as a result infrequently conducted; these results suggest, however, that it may be necessary to establish accurate associations between meteorological variables and waterborne disease. The need for pathogen-specific analyses was further underscored by the variability in seasonal peaks and general seasonal patterns, especially among pathogens within the same group. Seasonal variation can be a powerful tool for disentangling relationships between meteorological variables and infectious diseases because deviations from seasonal patterns can provide insight into the drivers of transmission [70, 71]. Given long time-series, even small changes in the seasonal environmental variables can indicate important, and potentially obscured, factors related to infectious disease dynamics. Extreme departures from seasonal norms, like rainfall during cyclonic storms, are also informative but their relative infrequency is limiting. Most waterborne diseases are considered highly seasonal, but we found considerable variability by geographic region and pathogen. Salmonellosis hospitalization rates peaked sharply in August in the Central Midwest but not in neighboring regions (North-Central Midwest, Mid-Atlantic) with similar meteorological seasonality. Conversely, in the North-Central Midwest *E*. *coli* hospitalizations peaked during the same time of year while Salmonellosis did not. More geographically and temporally resolved epidemiological data would allow a broader examination of why different regions exhibit distinct seasonality.

Bacterial and parasitic hospitalization rates were higher in small and rural hospitals and in Midwestern regions. Much of the Midwest experiences a wet spring season where the combination of snowmelt and intense precipitation can lead to flooding and heavy runoff [40]. Rural communities typically use drinking water from private wells, which are vulnerable to inundation during floods, or groundwater sources, which are often undertreated relative to surface drinking water [48, 49]. This is of particular concern in agricultural regions; both increased pathogen concentrations in water and illnesses have been associated with wet conditions near farms [45, 72].

The ability to persist in the environment or evade water treatment measures varies by pathogen and may help explain why the effect of meteorological conditions is not uniform. Only *Campylobacter* hospitalizations were significantly associated with environmental or drinking water variables. *Campylobacter* can enter a dormant state in the environment, persisting for weeks in water or sewage, but do not replicate outside of animal hosts [6, 73]. *Campylobacter* hospitalizations were positively associated with runoff, drinking water from groundwater sources, and rurality, results that are consistent with previous research identifying associations with precipitation, rural coastal areas, and untreated well water [6, 44].

Among the parasitic pathogens, we found that *Cryptosporidium* hospitalizations increased with average monthly precipitation. *Giardia* is a cyst-forming parasite but unlike *Cryptosporidium* (which forms oocysts), hospitalizations were not associated with environmental variables and demonstrated no discernable seasonality. The difference between these pathogens underscores the roles of pathogen biology and water treatment in transmission dynamics. While both pathogens colonize livestock and have been positively associated with wet conditions [74], they differ in their persistence in the environment and response to water treatment [22]. *Giardia* has been associated with high flowrates, indicating that runoff and flood conditions dilute its concentration and flush it out of the environment [50, 75]; *Giardia* is also easily removed from water, so treatment is highly effective [50]. *Cryptosporidium*, however, persists in water, potentially as part of biofilm communities [76, 77], and is highly resistant to chlorination [22].

Waterborne diseases have also been associated with drought conditions when pathogens are concentrated in diminished waterbodies; we found some evidence for this in hospitalizations for *E*. *coli*, which increased in months with lower precipitation, though this association was marginally significant. *E*. *coli* cases have been found to increase during dry periods and, in particular, during intense precipitation with antecedent dry periods [32].

Biofilm-forming bacteria may be an important source of community-acquired pneumonia (CAP) but their transmission dynamics outside hospital environments have not been thoroughly examined. We found hospitalizations for biofilm-related infections were positively associated with soil moisture, which integrates rainfall and snowmelt and reflects more extreme hydrological conditions including floods and droughts [78, 79]. Prolonged wet periods and overland flow likely mobilize these pathogens that naturally inhabit soil. The grouplevel association was driven by Legionnaires’ disease and respiratory *Pseudomonas*-related hospitalizations, though the importance of environmental drivers on transmission differed between them. The effect of soil moisture on Legionnaires’ disease was 10-times stronger compared to the group and while Legionnaires’ hospitalizations demonstrated consistent seasonality across geographic regions, there was no seasonality to *Pseudomonas* hospitalizations. This suggests that respiratory *Pseudomonas* is less tightly coupled to environmental variability, though this finding may be due to the inability to distinguish community-acquired and nosocomial infections.

Legionnaires’ disease hospitalization rates were higher in urban areas and in places that used drinking water from surface water sources; these associations were not statistically significant in the model framework but provide important guidance for future research with more temporally or geographically resolved data. Cities have complex distribution systems and a large number of premise plumbing systems that provide locations (e.g. pipes, holding tanks) for biofilm formation [12]. Rural drinking water sources are still vulnerable to contamination but non-centralized systems, and private wells in particular, offer fewer opportunities for biofilms to form or grow. Intestinal *Pseudomonas* hospitalizations, however, were substantially higher in rural areas and associated with precipitation at marginally significant levels; these associations closely mirror those of *Cryptosporidium*, and suggest that both infections share similar transmission mechanisms. The similarity between intestinal *Pseudomonas* and *Cryptosporidium*, in addition to the overall inconsistency between pathogen group-level and pathogen-specific findings, demonstrate the complexity of factors that influence waterborne transmission and indicate that they may not be adequately captured by broad categorization.

Our findings are constrained by several limitations. Hospitalizations only capture the most severe cases, which disproportionately occur among vulnerable groups, and represent a minority of all waterborne infections [1]. The seasonality of hospitalizations described here, however, is consistent with previous findings on the seasonality of cases and outbreaks; this suggests that the effect of meteorological conditions does not vary between hospitalized and non-hospitalized cases, though there are differences between individuals in these groups. The association between meteorological variables and non-hospitalized cases may be more pronounced, given that people who develop mild infections may have better overall health with few underlying conditions, and therefore would have been more likely to engage in recreational activities that can lead to exposure to contaminated water (e.g. fishing, swimming). The consistency of these findings should be assessed in future research that includes non-hospitalized cases. Our seasonality assessment was also limited to qualitative descriptions due to the length of the hospitalization data; longer time series for both hospitalizations and cases are necessary to rigorously quantify seasonality and how it varies among pathogens and regions.

The monthly resolution of the hospitalization data prevented examination of the effect of rapid changes in meteorological conditions, which may increase contamination by concentrating and then flushing pathogens [31, 32]. Data geolocation also introduces the potential for misclassification bias, given that meteorological data were associated with hospital locations, which may not reflect conditions at patients’ work and home. We aimed to address these limitations by repeating the study using hospital catchment areas as a sensitivity analysis, which was consistent with the primary findings. The primary limitation that could not be assessed with sensitivity analyses was the absence of water quality data, which is necessary to establish mechanistic associations between meteorological conditions and cases. Without water quality or individual-level epidemiological data, the probable route of exposure cannot be determined for hospitalized cases. The enteric bacteria are predominantly foodborne and many of these cases were likely infected through this transmission route rather than contaminated water. Future research should incorporate epidemiological surveillance, drinking water quality, and environmental water data to further examine the associations among meteorological variables and waterborne disease. Finally, the analysis also does not include data from the Southeast because these states did not report monthly data to HCUP; this is a major limitation as many Southeastern states include agricultural regions and experience substantial flooding associated with cyclonic storms.

The severity of floods and droughts are likely to change in conjunction with atmospheric warming; identifying the effect of environmental factors on waterborne infectious diseases is necessary to prepare for these events. Future research should aim to develop a comprehensive mechanistic model of contamination events by incorporating water quality data from environmental and drinking water sources. Detailed microbiological data would enable an exploration of the interactions of waterborne pathogens in water with multiple contaminants. In lab studies, biofilm formation was enhanced in water with biofilm-forming bacteria (*Legionella* and nontuberculous mycobacteria) and amoebas [80]; there were too few amoebic hospitalizations in this dataset to assess their relationship with biofilm-related infections, but in the future associations between microbiological contamination and infections should be examined. As most cases of waterborne disease are not hospitalized, future work should also expand to include all reportable cases; this is particularly important for understanding the burden of community-acquired pneumonia due to biofilm-forming pathogens. Some previous studies have found associations between waterborne diseases and extreme climatic events, including floods and droughts. These potential nonlinear effects are not captured in this analysis, and future work should examine cases and outbreaks due to extreme events.

## Supplementary Material

Supplemental Materials S1 Table. Average hospitalizations per 10,000 annual discharges by hospital location and type.
S2 Table. Average monthly hospitalizations per 10,000 annual discharges by drinking water variables for the pathogen groups.
S3 Table. Description of the hospitals included in the analysis by pathogen group using HCUP variables and drinking water source data.
S4 Table. Description of the hospitals by specific pathogen using HCUP variables and drinking water source data.
S5 Table. Assessment of time series trends by pathogen group using Mann-Kendall test.
S1 Text. Description of the generalized linear mixed model (GLMM).

Figure S6 **S6 Fig. Best model effect estimates for each pathogen group across different case-count thresholds.** As a sensitivity analysis, the data were restricted use 5-, 10-, 15-, and 20-case thresholds as cutoffs for inclusion in the hospitalization dataset. The effect estimates were consistent across the case-count thresholds.

Figure S5 **S5 Fig. Time series for pathogen-group hospitalizations by drinking water source.** After 2006, biofilm-related hospitalizations increased in areas served by surface water and decreased in areas that used groundwater for drinking water.

Figure S4 **S4 Fig. Time series for biofilm-related hospitalizations per 10,000 discharges averaged by geographic regions.** Hospitalizations for Legionnaires’ disease and NTM increased between 2000 and 2011 in New England, Mid-Atlantic, and Midwestern hospitals.

Figure S3 **S3 Fig. Seasonality of biofilm-related hospitalizations by geographic region.** Average monthly Legionnaires’ disease hospitalizations per 10,000 discharges peaked in New England and Mid-Atlantic states earlier in the year (July–August) compared to Midwestern and Mountain states (September–October). The other respiratory biofilm-forming pathogens exhibited no discernible seasonality. Intestinal *pseudomonas* hospitalizations peaked between October and February in some regions but there were few hospitals in the intestinal *Pseudomonas*-specific dataset.

Figure S2 **S2 Fig. Seasonality of parasitic hospitalizations by geographic region.** Average monthly *Cryptosporidium* hospitalizations per 10,000 discharges in the Midwestern regions were the only parasitic hospitalizations to show strong seasonality. *Giardia* hospitalizations in the Pacific region also demonstrated a seasonal peak in August, but there were few hospitalizations in that area.

Figure S1 **S1 Fig. Seasonality of bacterial hospitalizations by geographic region.** Average monthly hospitalizations per 10,000 discharges for all bacterial pathogens exhibited clear seasonality, with most peaking in the late summer or early fall. *Campylobacter* hospitalizations peaked earlier in the year compared to the other bacterial pathogens.

## Figures and Tables

**Table 1.** Total count of hospitalizations for pathogen groups and specific pathogens or diseases between 2000 and 2011.

Pathogen group	Specific pathogen or disease	Number of cases	Percent of all hospitalizations	Est. percent waterborne [1]
**Enteric bacteria**		9, 259	16.1	
	*Salmonella*	4,587	8.0	6
	*Shigella*	1,024	1.8	4
	*E. coli*	1,451	2.5	5^a^, 6^b^
	*Campylobacter*	2,197	3.8	13
**Parasite**		1,580	2.7	
	*Giardia*	654	1.1	44
	*Cryptosporidium*	661	1.2	43
	Protozoa	79	0.1	-
	Amoeba	186	0.3	-
**Biofilm-forming bacteria**		46,221	80.7	
	Legionnaires’ disease	2,327	4.1	97
	Respiratory *pseudomonas*	37,681	65.7	51
	Intestinal *pseudomonas*	717	1.3	-
	Nontuberculous mycobacteria (NTM)	5,496	9.6	72
**Virus**				
	Norovirus	275	0.4	6

a Estimates for STEC infection, serotype O157

b Estimates for STEC infections, serotype non-O157

**Table 2.** Average monthly hospitalization rates per 10,000 discharges by hospital location and type for the pathogen groups.

Hospital characteristics^a^	Bacteria Mean ± SD	P	Biofilm-forming Mean ± SD	P	Parasite Mean ± SD	P	Virus Mean ± SD	P
**Number of hospitals**	302		516		89		13	
**Hospital bed size**								
**Small**	0.35 (1.02)		2.6 (14.81)		0.26 (1.02)		0.32 (0.58)	
**Medium**	0.29 (0.94)		0.89 (2.04)		0.17 (0.61)		0.77 (5.88)	
**Large**	0.23 (0.55)	<0.001	0.73 (1.45)	<0.001	0.1 (0.27)	<0.001	0.14 (0.64)	0.24
**Hospital location**								
**Rural**	0.48 (1.42)		1.33 (3.49)		0.35 (1.23)		1.06 (8.39)	
**Urban**	0.22 (0.50)	<0.001	1.13 (8.01)	<0.001	0.11 (0.38)	0.41	0.24 (0.89)	0.001
**Region**								
**New England**	0.21 (0.51)	0.26	0.70 (1.34)	<0.001	0.08 (0.17)	0.90	-	
**Mid-Atlantic**	0.29 (0.86)	0.37	1.16 (5.38)	<0.001	0.16 (0.61)	0.96	0.50 (4.66)	0.52
**Central Midwest**	0.25 (0.70)	-	1.41 (9.81)	-	0.12 (0.34)	-	0.21 (0.84)	-
**North-Central Midwest**	0.35 (1.02)	<0.001	2.6 (14.81)	<0.001	0.26 (1.02)	0.05	0.32 (0.58)	0.24
**Mountain**	0.29 (0.94)	<0.001	0.89 (2.04)	<0.001	0.17 (0.61)	0.001	0.77 (5.88)	0.24
**Pacific**	0.23 (0.55)	-	0.73 (1.45)	-	0.1 (0.27)	-	0.14 (0.64)	-
**Water source**								
**Groundwater**	0.30 (0.88)		1.39 (10.16)		0.17 (0.70)		0.55 (5.62)	
**Surface water**	0.24 (0.68)	0.13	1.06 (4.47)	<0.001	0.11 (0.31)	0.97	0.28 (0.99)	0.26

a Differences between or among hospital types were assessed for each pathogen group using Kruskal-Wallis test (for multiple groups) and Mann-Whitney U test (two groups) for non-parametric continuous data.

**Table 3.** Associations between hospitalization rates and meteorology, drinking water source, and location in top models identified by multimodel inference.

Pathogen group	Precipitation	Soil moisture	Runoff	Temperature	Surface water	Urban
Enteric bacteria	-	0.09 (−0.02, 0.21)		-	-	**−0.16 (−0.24, −0.08)**
*Salmonella*	-	0.09 (−0.09, 0.28)	-		0.01 (−0.12, 0.15)	-
*Shigella*	0.04 (−0.13, 0.21)	-	-		−0.14 (−0.65, 0.36)	-
*E*. *coli*	−0.14 (−0.29, 0.01)	-	-		-	−0.15 (−0.49, 0.18)
*Campylobacter*	-	-	**0.11 (0.04, 0.17)**		**−0.27 (−0.48, −0.06)**	**−0.31 (−0.53, −0.09)**
Biofilm-forming bacteria	-	**0.12 (0.07, 0.17)**		-	0.05 (−0.03, 0.14)	-
**Legionnaires’ disease**	-	**1.24 (0.9, 1.57)**	-		-	0.16 (−0.04, 0.37)
**NTM**	-	−0.09 (−0.25, 0.07)	-		−0.17 (−0.38, 0.03)	-
Respiratory *pseudomonas*	-	**0.09 (0.04, 0.15)**	-		0.08 (−0.01, 0.17)	-
Intestinal *pseudomonas*	0.16 (−0.11, 0.42)	-	-		-	**−0.62 (−1.21, −0.02)**
Parasite	**0.09 (0, 0.18)**	-		-	−0.1 (−0.3, 0.09)	-
*Cryptosporidium*	**0.22 (0.01, 0.44)**	-			-	−0.33 (−0.83, 0.17)
*Giardia*	−0.01 (−0.2, 0.19)	-	-		-	0.08 (−0.44, 0.59)
**Protozoa**	-	-	−0.47 (−1.92, 0.98)		-	-
**Amoeba**	0.24 (−0.02, 0.51)	-	-		-	-
Virus	-	-		−15.7 (−37.2, 5.71)	-	-

Note: statistically significant effect estimates and 95% CI are in bold text.

## Data Availability

We are unable to share the minimal dataset underlying the results because it contains potentially identifying information and cannot be publicly shared per the Healthcare Cost and Utilization Project (HCUP) Data Use Agreement (DUA) (https://hcup-us.ahrq.gov/team/NationwideDUA.pdf). The National Inpatient Sample (NIS) datasets from HCUP are publicly and easily available to purchase after completion of the Data Use Agreement Training Course (https://hcup-us.ahrq.gov/tech_assist/centdist.jsp). The contact information for requesting the data from the HCUP Central Distributor is: HCUP-RequestData@ahrq.gov; (866) 556–4287 (phone); (866) 792–5313 (fax).
