# Supplementary material for: Hydrometeorology and geography affect hospitalizations for waterborne infectious diseases in the United States: A retrospective analysis
Source: PLOS Water. Author manuscript; Available in PMC 2025 Aug 29. (PMC12392087; doi:10.1371/journal.pwat.0000206)
Supplement: Supplemental Materials — S1 Table. Average hospitalizations per 10,000 annual discharges by hospital location and type. S2 Table. Average monthly hospitalizations per 10,000 annual discharges by drinking water variables for the pathogen groups. S3 Table. Description of the hospitals included in the analysis by pathogen group using HCUP variables and drinking water source data. S4 Table. Description of the hospitals by specific pathogen using HCUP variables and drinking water source data. S5 Table. Assessment of time series trends by pathogen group using Mann-Kendall test. S1 Text. Description of the generalized linear mixed model (GLMM). [file NIHMS2093782-supplement-Supplemental_Materials.pdf]

Supporting Information

Tables

S1 Table. Average hospitalizations per 10,000 annual discharges by hospital location and type

Pathogen	Salm.	Shigella	E. coli	Campy.	Crypto.	Giardia	Protozoa	Amoeba	Legionnaires' disease	NTM	Pseudomonas	Int. pseudo	Norovirus
No. cases	4,587	1,024	1,451	2,197	661	654	79	186	2,327	5,496	37,681	717	275
No. hospitals	173	26	40	71	20	27	2	11	75	147	496	9	13
Hospital Location(%)													
Rural	0.306	0.069	0.125	0.144	0.338	0.092	0	0.003	0.053	0.075	1.716	0.049	1.058
Urban	0.153	0.038	0.055	0.085	0.068	0.062	0.007	0.015	0.07	0.511	1.495	0.029	0.24
Hospital Bedsize (%)													
Small	0.184	0.055	0.088	0.153	0.215	0.111	0.004	0.006	0.076	1.105	2.583	0.055	0.323
Medium	0.19	0.053	0.067	0.102	0.102	0.08	0.005	0.014	0.057	0.114	1.288	0.027	0.766
Large	0.178	0.037	0.064	0.079	0.059	0.05	0.007	0.015	0.062	0.135	1.176	0.031	0.139
Region (%)													
New England	0.174	0.023	0.062	0.124	0.047	0.057	0.006	0.006	0.097	0.144	1.188	0.031	0.216
Mid-Atlantic	0.22	0.033	0.036	0.078	0.051	0.056	0.004	0.011	0.095	0.143	1.547	0.021	-
Central Midwest	0.184	0.048	0.105	0.079	0.074	0.086	0	0.006	0.062	0.06	2.269	0.085	-
North-Central Midwest	0.159	0.04	0.112	0.132	0.203	0.07	0.003	0.007	0.049	0.089	1.184	0.035	0.138
Mountain	0.173	0.071	0.085	0.072	0.031	0.059	0.016	0.023	0.049	3.03	1.156	0.032	-
Pacific	0.153	0.061	0.061	0.097	0.041	0.074	0.011	0.033	0.031	0.113	1.834	0.032	0.7
Water Source (%)													
Groundwater	0.186	0.05	0.086	0.114	0.146	0.055	0.005	0.012	0.055	0.756	1.393	0.039	0.553
Surface water	0.181	0.045	0.059	0.087	0.052	0.068	0.007	0.015	0.071	0.127	1.675	0.033	0.281

S2 Table. Average monthly hospitalizations per 10,000 annual discharges by drinking water variables for the pathogen groups

CWS characteristics[a]	Bacteria	Biofilm-forming pathogens	Parasite	Virus
Water Source				
Groundwater	0.299*	0.977	0.168	0.553
Surface water	0.244	1.066*	0.110	0.281
Ownership of Water System				
Federal	0.207	0.582	0.129	-
Local	0.264	0.961	0.135	0.423
Private/public	0.161	0.393	0.044	-
Native American	0.273	0.673	-	-
Private	0.321*	1.831*	0.086	0.225
State	0.202	1.536*	0.035	-
Primary Water Source				
Groundwater infl. by surface water	0.392	1.001	0.179	-
Purchased groundwater infl. by surface water	0.248	0.702	-	-
Groundwater	0.302	0.945	0.168	0.609
Purchased groundwater	0.158	1.842*	-	0.233
Surface water	0.240	0.986	0.101	0.411
Purchased surface water	0.237	1.182	0.118	0.105
Water Source Protection				
Water source protection not implemented	0.254	1.017*	0.115	0.205
Water source protection implemented	0.211	0.696	0.076	-
Not reported	0.289	1.171	0.158	0.503

[a]Differences between or among hospital types were assessed for each pathogen group using Kruskal-Wallis test (for multiple groups) and Mann-Whitney U test (two groups) for non-parametric continuous data.

*Indicates significant differences between or among CWS characteristics ($p < 0.05$).

S3 Table. Description of the hospitals included in the analysis by pathogen group using HCUP variables and drinking water source data

**Hospital characteristics**[a]	Bacteria	Parasites	Biofilm-forming bacteria	Virus	Overall
No. of hospitals	302	89	516	13	524
Hosp. Location (%)					
Rural	19.2	6.2	31.7	20	24.9
Urban	80.8	93.8	68.3	80	75.1
Hospital Bedsize (%)					
Small	13.9	8.8	25.2	1.7	19.5
Medium	30.2	26.9	30.6	41.7	30.2
Large	55.9	64.3	44.2	56.7	50.3
Region (%)					
New England	10	10.2	7.9	28.3	9.1
Mid-Atlantic	32.6	34.3	30.1	0	30.9
Central Midwest	7.5	5.5	12.6	0	10
North-Central Midwest	19	23.8	19.4	28.3	19.8
Mountain	10.6	13.1	8.9	0	9.8
Pacific	20.3	13.1	21.1	43.3	20.3
Water Source (%)					
Groundwater	33.2	38.2	34.4	46.2	36.3
Surface water	66.8	61.8	65.6	53.8	63.7
Annual Discharge					
Mean (SD)	16,100 (11,100)	24,100 (13,500)	11,300 (10,600)	17,800 (10,700)	14,200 (11,800)
Median (Min, Max)	14,000 (1,040, 65,800)	23,100 (1,500, 65,800)	7,670 (76, 65,800)	17,100 (503, 42,600)	10,900 (76, 65,800)

[a]Differences between or among hospital types were assessed for each pathogen group using Kruskal-Wallis test (for multiple groups) and Mann-Whitney U test (two groups) for non-parametric continuous data. There were significant differences by hospital characteristics ($p < 0.05$) for all of the categories except water source. Differences among the pathogen groups, however, were mostly insignificant.

S4 Table. Description of the hospitals by specific pathogen using HCUP variables and drinking water source data

Pathogen	Salm.	Shigella	E. coli	Campy.	Crypto.	Giardia	Protozoa	Amoeba	Legionnaires' disease	NTM	Pseudomonas	Int. pseudo	Norovirus
No. cases	4,587	1,024	1,451	2,197	661	654	79	186	2,327	5,496	37,681	717	275
No. hospitals	173	26	40	71	20	27	2	11	75	147	496	9	13
Hospital Location (%)													
Rural	13.6	5	14.4	4.9	9.8	0	0	2.9	6.4	5.6	30.4	14.6	20
Urban	86.4	95	85.6	85.1	90.2	100	100	97.1	93.6	94.4	69.6	85.4	80
Hospital Bedsize (%)													
Small	9.1	9.3	6.7	6.1	10.8	4.4	0	0	9.8	9.3	24.5	0	1.7
Medium	23.2	30	17.4	25	15.7	19	0	18.8	24.9	26.5	31	39	41.7
Large	67.6	60.7	75.9	68.9	73.5	76.6	100	81.2	65.4	64.1	44.6	61	56.7
Region (%)													
New England	9	9.3	17.4	12.8	5.9	10.2	64.3	0	14	10.7	8.2	14.6	28.3
Mid-Atlantic	41.3	28.6	24.6	29.4	37.3	40.9	0	31.9	52.2	39	30.3	31.7	0
Central Midwest	6	3.6	6.7	1.2	4.9	6.6	0	0	5.9	4.1	12.7	9.8	0
North-Central Midwest	14.8	9.3	23.1	19.2	38.2	19.7	0	7.2	10.9	11.4	19.4	12.2	28.3
Mountain	9.6	22.9	16.9	12.5	4.9	13.9	35.7	11.6	12.3	14.1	8.1	22	0
Pacific	19.2	26.4	11.3	25	8.8	8.8	0	49.3	4.7	20.7	21.3	9.8	43.3
Water Source (%)													
Groundwater	32	30.8	27.5	29.6	47.6	25	0	100	35.5	27.2	37.5	22.2	46.2
Surface water	68	69.2	72.5	70.4	52.4	75	100	0	64.5	72.8	62.5	77.8	53.8

Primary Water Source													
GW	30.9	30.8	27.5	29.6	47.6	25		100	35.5	27.2	35.9	22.2	38.5
GU	1.7	0	2.5	2.8	4.8	0		0	0	1.3	1.8	11.1	0
GWP	36.6	34.6	42.5	36.6	33.3	50		0	36.8	39.1	33.9	55.6	30.8
SW	29.1	34.6	27.5	31	14.3		0	27.6	32.5	26.3	0	23.1	
SWP	0.6	0	0	0	0		0	0	0	0.4	11.1	0	<0.001
Annual Discharge													
Mean (SD)	20,500 (11,800)	25,100 (15,000)	24,700 (15,200)	23,200 (13,000)	28,100 (18,000)	37,400 (4,950)	25,700 (12,800)	23,300 (13,100)	21,600 (11,600)	11,600 (10,700)	18,900 (15,300)	17,800 (10,700)	
Median (Min, Max)	19,200 (2,130, 65,800)	24,600 (1,570, 64,700)	22,100 (4,320, 65,800)	21,700 (2,170, 64,700)	25,000 (3,610, 65,800)	36,300 (28,600, 43,500)	23,900 (6,170, 65,800)	20,800 (1,760, 65,800)	20,100 (76, 65,800)	8,080 (128, 65,800)	15,500 (4,500, 65,800)	17,100 (503, 42,600)	

S5 Table. Assessment of time series trends by pathogen group using Mann-Kendall test

Pathogen group	Mann-Kendall p-value	Mann-Kendall slope	Mann-Kendall indicator
Bacteria	0.338	7.01E-06	0.429
Biofilm	0.613	6.14E-04	0.744
Parasite	0.685	6.76E-06	1
Virus	<0.001	8.89E-04	2.34

Supplementary models

S1 Model. Description of the generalized linear mixed model (GLMM)

The observed number of hospitalizations for a given waterborne disease pathogen group in hospital *i* at month *t*, Y_{it}, is assumed to be distributed as a negative binomial variable:

$$Y_{it} \sim NB\ (Y_{it} \mid \mu_{it}, \theta)$$

where μ_{it} is the mean, θ is the shape parameter and $t = 1,\ldots, n$. Here *n* (144) is the number of months in the study period.

In the multimodel inference approach, the following model is run with every combination of meteorological, drinking water source, hospital location, and geographic region variables for each waterborne disease pathogen group.

The model structure is:

$$\log(\mu_{it}) = X_{it}\beta + Z_{it}b + \log(P_{it})$$

Where X_{it} includes the predictor variables assessed in multimodel inference in addition to monthly sine and cosine terms to account for seasonality ($\sin(2\pi \text{ x } \frac{\text{month}_t}{12})$ and $\cos(2\pi \text{ x } \frac{\text{month}_t}{12})$), and a term for year to account for long-term trends. β is the vector of fixed effects for X_{it}. Z_{it} is the matrix for *k* random effects, where *k* is the number of hospitals in the study, and b is the *k* x 1 vector of the random effects. P_{it} is the population offset, which is the total, all-cause monthly hospitalizations for hospital *i* in time *t*. The vector of *k* random effects, b, is assumed to follow a normal distribution: $b \sim N_k\ (0, \Psi)$ where Ψ is a positive definite variance-covariance matrix that determines the random effects.

The specific predictor variables assessed in via multimodel inference are precipitation, soil moisture, runoff, and temperature as continuous meteorological variables; groundwater or surface water as a binary drinking water source variable; urban or rural as a binary hospital location variable; and geographic region as a categorical variable (New England, Mid-Atlantic, Central Midwest, North-Central Midwest, Mountain, Pacific).

Figure captions

S1 Figure. Seasonality of bacterial hospitalizations by geographic region. Average monthly hospitalizations per 10,000 discharges for all bacterial pathogens exhibited clear seasonality, with most peaking in the late summer or early fall. *Campylobacter* hospitalizations peaked earlier in the year compared to the other bacterial pathogens.

S2 Figure. Seasonality of parasitic hospitalizations by geographic region. Average monthly *Cryptosporidium* hospitalizations per 10,000 discharges in the Midwestern regions were the only parasitic hospitalizations to show strong seasonality. *Giardia* hospitalizations in the Pacific region also demonstrated a seasonal peak in August, but there were few hospitalizations in that area.

S3 Figure. Seasonality of biofilm-related hospitalizations by geographic region. Average monthly Legionnaires' disease hospitalizations per 10,000 discharges peaked in New England and Mid-Atlantic states earlier in the year (July – August) compared to Midwestern and Mountain states (September – October). The other respiratory biofilm-forming pathogens exhibited no discernible seasonality. Intestinal *pseudomonas* hospitalizations peaked between October and February in some regions but there were few hospitals in the intestinal *Pseudomonas*-specific dataset.

S4 Figure. Time series for biofilm-related hospitalizations per 10,000 discharges averaged by geographic regions. Hospitalizations for Legionnaires' disease and NTM increased between 2000 and 2011 in New England, Mid-Atlantic, and Midwestern hospitals.

S5 Figure. Time series for pathogen-group hospitalizations by drinking water source. After 2006, biofilm-related hospitalizations increased in areas served by surface water and decreased in areas that used groundwater for drinking water.

S6 Figure. Best model effect estimates for each pathogen group across different case-count thresholds. As a sensitivity analysis, the data were restricted use 5-, 10-, 15-, and 20-case thresholds as cutoffs for inclusion in the hospitalization dataset. The effect estimates were consistent across the case-count thresholds.
