# Supplementary figures and images for: Hydrometeorology and geography affect hospitalizations for waterborne infectious diseases in the United States: A retrospective analysis
Source: PLOS Water. Author manuscript; Available in PMC 2025 Aug 29. (PMC12392087; doi:10.1371/journal.pwat.0000206)

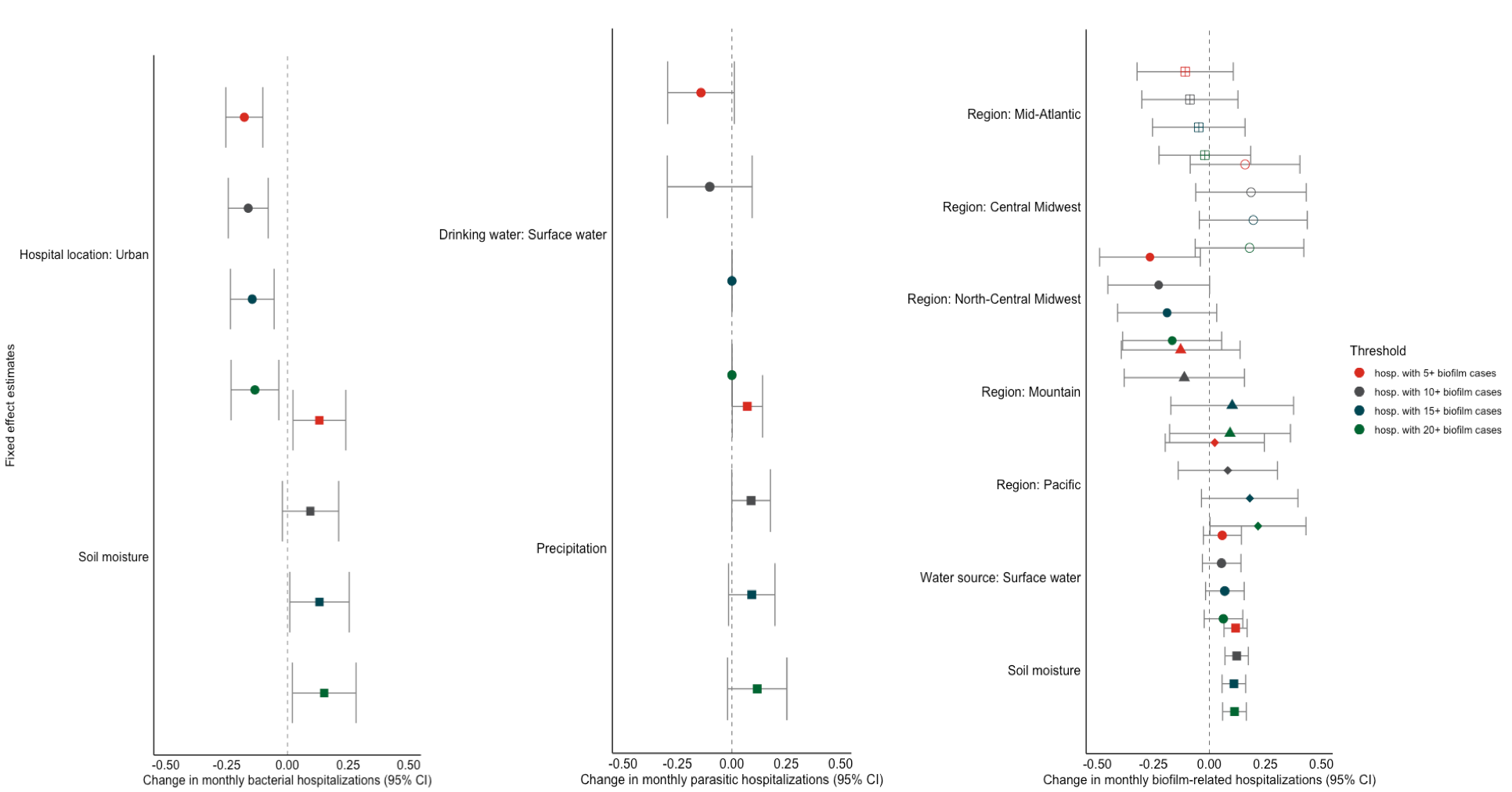

Supplement: Figure S6 — S6 Fig. Best model effect estimates for each pathogen group across different case-count thresholds. As a sensitivity analysis, the data were restricted use 5-, 10-, 15-, and 20-case thresholds as cutoffs for inclusion in the hospitalization dataset. The effect estimates were consistent across the case-count thresholds. [file NIHMS2093782-supplement-Figure_S6.tif]

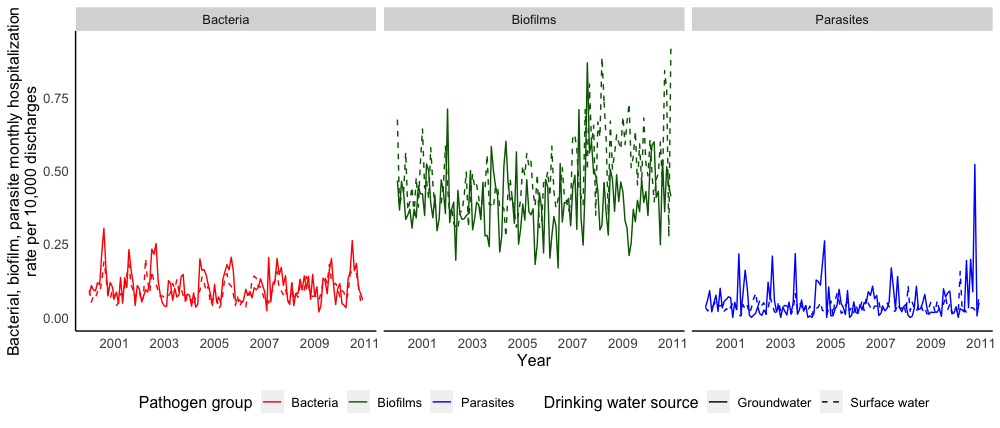

Supplement: Figure S5 — S5 Fig. Time series for pathogen-group hospitalizations by drinking water source. After 2006, biofilm-related hospitalizations increased in areas served by surface water and decreased in areas that used groundwater for drinking water. [file NIHMS2093782-supplement-Figure_S5.tiff]

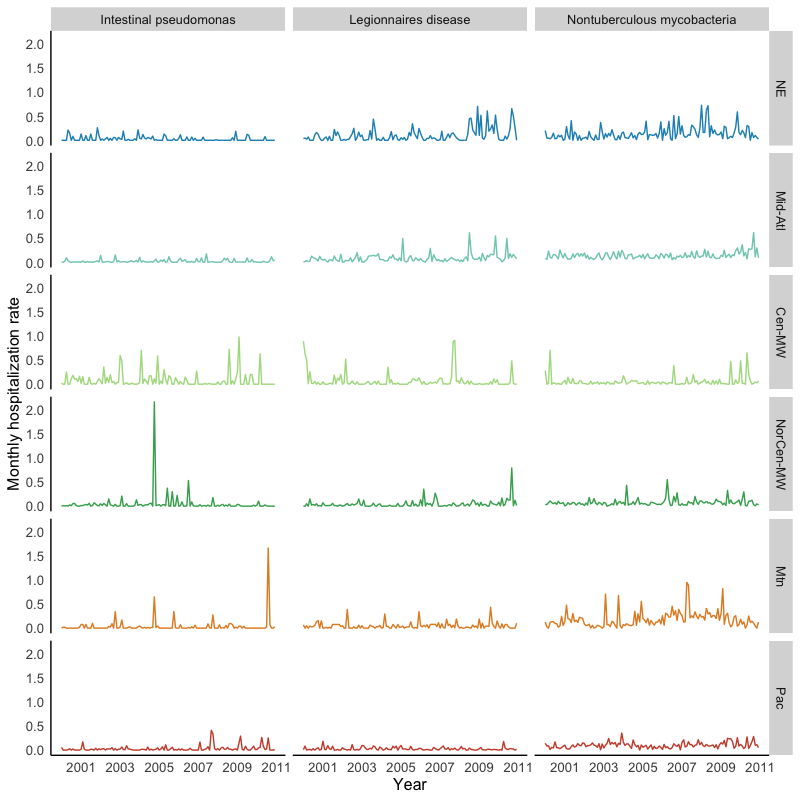

Supplement: Figure S4 — S4 Fig. Time series for biofilm-related hospitalizations per 10,000 discharges averaged by geographic regions. Hospitalizations for Legionnaires’ disease and NTM increased between 2000 and 2011 in New England, Mid-Atlantic, and Midwestern hospitals. [file NIHMS2093782-supplement-Figure_S4.tiff]
